# Acquiring of knowledge about sexual health by blind people: an action research*

**DOI:** 10.1590/1518-8345.3006.3163

**Published:** 2019-07-18

**Authors:** Inacia Sátiro Xavier de França, Alexsandro Silva Coura, Francisco Stélio de Sousa, Jamilly da Silva Aragão, Arthur Felipe Rodrigues Silva, Sérgio Ribeiro dos Santos

**Affiliations:** 1 Universidade Federal da Paraíba, João Pessoa, PB, Brasil; 2 Universidade Estadual da Paraíba, Campina Grande, PB, Brasil; 3 Universidade de Pernambuco, Recife, PE, Brasil; 4 Bolsista da Fundação de Amparo a Ciência e Tecnologia do Estado de Pernambuco (FACEPE), Brasil; 5 Bolsista da Coordenação de Aperfeiçoamento de Pessoal de Nível Superior (CAPES), Brasil

**Keywords:** Nursing, Sexual Health, Blindness, Sexually Transmitted Diseases, Knowledge, Health Education, Enfermagem, Saúde Sexual, Cegueira, Doenças Sexualmente Transmissíveis, Enfermería, Salud Sexual, Ceguera, Enfermedades de Transmisión Sexual, Conocimiento, Educación em Salud

## Abstract

**Objective:**

to evaluate knowledge about sexual health, with blind people, before and after educational intervention.

**Method:**

action research conducted with 58 blind people enrolled in a philanthropic educational institution. A form with sociodemographic and knowledge variables about Sexually Transmitted Infections was used. The Chi-square and Fisher tests were performed.

**Results:**

men presented higher frequency of alcoholism (p <0.001) and illicit drugs (p = 0.006). It was found that they used a male condom more frequently than women using a female condom (p = 0.003), although they had more knowledge about the prevention of Sexually Transmitted Infections (p = 0.006). Among these infections, Trichomonas vaginalis (52.4%) was more frequent. Knowledge gaps on risk factors and safe sex were identified. After the intervention, an increase in the knowledge about sexual health was detected.

**Conclusion:**

the educational intervention, in the light of problematizing pedagogy, (re) constructed the knowledge on sexual health, empowering the participants regarding the prevention of Sexually Transmitted Infections. Therefore, it is necessary that nurses carry out educational interventions with this clientele, aiming to soften deficits of knowledge about the thematic in screen.

## Abstract (pt)

**Objetivo:**

avaliar o conhecimento sobre a saúde sexual, junto a pessoas cegas, antes e depois de intervenção educativa.

**Método:**

pesquisa-ação realizada com 58 pessoas cegas matriculadas em instituição educacional filantrópica. Utilizou-se formulário com variáveis sociodemográficas e de conhecimento sobre Infecções Sexualmente Transmissíveis. Efetuaram-se os testes de Qui-quadrado e Fisher.

**Resultados:**

os homens apresentaram maior frequência de etilismo (p<0,001) e drogas ilícitas (p=0,006). Constatou-se que estes usavam preservativo masculino com mais frequência em relação às mulheres com o uso do preservativo feminino (p=0,003), embora estas apresentassem maior conhecimento acerca das formas de prevenção das Infecções Sexualmente Transmissíveis (p=0,006). Dentre essas infecções, verificou-se, como mais frequente, a *Trichomonas vaginalis* (52,4%). Identificaram-se lacunas do conhecimento sobre fatores de risco e sexo seguro. Após a intervenção, detectou-se a ampliação do conhecimento sobre saúde sexual.

**Conclusão:**

a intervenção educativa, à luz da pedagogia problematizadora, (re) construiu o conhecimento sobre saúde sexual, empoderando os participantes quanto à prevenção de Infecções Sexualmente Transmissíveis. Portanto, é mister que enfermeiros executem intervenções educativas para com esta clientela, visando a amenizar *deficits* de conhecimentos sobre a temática em tela.

## Abstract (es)

**Objetivo:**

evaluar el conocimiento sobre la salud sexual, junto a personas ciegas, antes y después de intervención educativa.

**Método:**

investigación-acción, realizada con 58 personas ciegas, matriculadas en institución educativa filantrópica. Se utilizó formulario con variables sociodemográficas y de conocimiento sobre Infecciones Sexualmente Transmisibles. Se realizaron las pruebas de Chi-cuadrado y Fisher.

**Resultados:**

los hombres presentaron mayor frecuencia de etilismo (p <0,001) y drogas ilícitas (p = 0,006). Se constató que éstos usaban preservativo masculino con más frecuencia en relación a las mujeres con el uso del preservativo femenino (p = 0,003), aunque éstas presentaban mayor conocimiento acerca de las formas de prevención de las Infecciones Sexualmente Transmisibles (p = 0,006). Entre estas infecciones, se verificó, como más frecuente, la *Trichomonas vaginalis* (52,4%). Se identificaron lagunas del conocimiento sobre factores de riesgo y sexo seguro. Después de la intervención, se detectó la ampliación del conocimiento sobre salud sexual.

**Conclusión:**

la intervención educativa, a la luz de la pedagogía problematizadora, (re) construyó el conocimiento sobre salud sexual, empoderando a los participantes en la prevención de Infecciones Sexualmente Transmisibles. Por lo tanto, es menester que los enfermeros ejecuten intervenciones educativas para con esta clientela, con el fin de suavizar los déficits de conocimientos sobre la temática en pantalla.

## Introduction

In Brazil, Sexually Transmitted Infections (STIs) are considered a serious public health problem, despite the existence of a national policy for the prevention and treatment of STI / Acquired Immunodeficiency Syndrome (AIDS). It is stated that in many health centers across the country there is a shortage of physicians and nurses adequately trained to care for patients with these infections^(^ ^1^ ^)^.

In addition to the exceptions and unpreparedness of some professionals, it is considered that the estimated incidence of human papillomavirus (HPV) infection is of the order of 685,400 new cases annually^(^ ^2^–^3^ ^)^. There is a need to intensify campaigns for clarification and prevention in relation to Chlamydia trachomatis and Neisseria gonorrhoeae and to the possible infertility due to these treatable infections, which mainly affect the young population of lower income^(^ ^4^ ^)^. Therefore, if the incidence or prevalence of STIs is high in a country or region, groups that are vulnerable to STIs, in the biopsychosocial and socio-cultural aspects, are at greater risk of sexual transmission by HIV^(^ ^5^ ^)^.

It is worth noting that, despite the prevention and treatment of STIs being practices inserted in family planning, the access of users with this type of infection is marked by minimal demand^(^ ^6^ ^)^. In this context, the segment of people with visual impairment is among the groups most vulnerable to health risks^(^ ^7^ ^)^, since visual loss entails disadvantages and restrictions on participation and performance in daily activities, impairing independence, the autonomy and the quality of life of people with this aggravation^(^ ^8^ ^)^.

These people usually narrate feelings of rejection or overprotection in the family, difficulties in acquiring orthoses and prostheses for autonomy, little investment in study and professional qualification, as well as confronting physical and attitudinal barriers in health services, as well as obstacles to the sexuality and motherhood^(^ ^9^ ^)^. It can be observed that stigmas about the sexuality of the population of people with disabilities are still present in the 21st century, with the belief that they cannot have children or even practice the sexual act^(^ ^7^ ^,^ ^9^ ^–^ ^10^ ^)^.

In the case of women with disabilities, the primary care services do not recognize the sexual and reproductive rights and the double vulnerability that affects them, a fact that causes the persistence of the profile of fragility in the health care of these people due to the disarticulation and discontinuity of actions in the public and private spheres and the low level of coverage of care^(^ ^9^ ^)^.

In the case of blind people, the sum of the myths and taboos about sexuality, their inherent vulnerability and the difficulties of access to health services, has repercussions on sexual and reproductive health, in view of exposure to the risk of contracting STIs. In this sense, it is indicated the possibility of occurrence of these infections by the disinformation about the prevention, since the health policies and the preventive campaigns are directed to the visionary public and are not adapted for this social segment, which increases its vulnerability^(^ ^7^ ^,^ ^9^ ^–^ ^11^ ^)^.

In this context, it is up to the Nursing professional to plan and execute health education actions, aiming to (re) build their knowledge about sexual and reproductive health and to prevent / reduce STI cases^(^ ^6^ ^)^. In this way, health education makes it possible to meet the information needs of users of the Unified Health System, considering that this action is based on the development of activities that enable the empowerment of people for self-care and health promotion^(^ ^12^ ^)^.

In view of the above, this study sought answers to the question: how blind people care for sexual health? It is considered imperative to carry out this research that can positively contribute to the Nursing intervention, through the use of health education, to promote the (re) construction of blind people's knowledge about sexual health.

This research has the potential to contribute to the actions of Nursing in the field of sexual health education for people with disabilities, since the report of the characterization of the vulnerability situation of these people enables managers and health professionals to plan strategies and actions of prevention in line with the social and health reality of these individuals. In addition, it is justified that the issue of people with disabilities can be found in the National Agenda for Priorities in Health Research.

In this perspective, the objective was to evaluate the knowledge about sexual health, together with blind people, before and after educational intervention.

## Method

A research-action study, with empirical basis and quantitative approach, held in Campina Grande, Paraíba, Brazil, from April 2014 to December 2016, at Campina Grande Institute of the Blind (ICCG), a philanthropic educational institution for blind people.

The population was 65 blind people who attended the activities of this institution. After the authorization of the institutional leadership, a meeting was held with the people assisted to present the research and the invitation to participate. The sample consisted of 58 blind people, using a random lottery and the formula: n = N. Z2. P (1-P) / (N-1). e2 + Z2. P (1-P), where: n = Sample value; N = Value of population; Z = confidence interval (1.96); P = Prevalence (50%) e = Tolerated error (0.05).

The inclusion criteria considered for the study were: minimum age of 18 years and attending, with assiduity, the activities of the institution. Persons not residing in the urban area of Campina Grande, Paraíba, Brazil were excluded.

For the implementation of the educational intervention of Nursing, the problematizing pedagogy^(^ ^13^–^15^ ^)^ was used, adapted for this study and developed in the following stages:

- Thematic research and survey of the generating themes, applying to the participants a closed questionnaire about sociodemographic and open conditions about knowledge about STIs, risk factors, safe sex, consequences of STIs for reproduction and self-reported STIs;
- Encoding and decoding of the answers obtained with the form for the apprehension of the knowledge / ignorance of the participants in relation to the information specified in the open questions. The clipping of problem situations was based on the themes of the educational action, which was developed on a scheduled day and time, in agreement with the participants, according to the availability of the group. Three meetings were held that took place once a week, lasting two hours. At each meeting, a specific theme was addressed: 1 - STI, forms of transmission and possible sequels resulting from STIs; 2 - Signs and symptoms of STI / AIDS; 3 - How to prevent STI / AIDS;
- Critical disclosure, which occurred three months after the educational action, reapplying the form, but only with the open questions about knowledge about STIs, risk factors, safe sex and consequences of STI for reproduction, aiming to evaluate the result post-educational intervention.

The educational intervention was developed by a master's level nurse, who also coordinated a team of nursing undergraduate students, duly trained to perform the data collection, before and after the intervention.

Data was then processed in the Statistical Package for the Social Sciences (SPSS), version 20.0, and analyzed using descriptive statistics, using absolute and relative frequencies, as well as analytical statistics, hypothesis. To verify the existence of an association between health variables and prevention of STIs with sex, the Chi-square test was used, which was also used to compare the observed number related to knowledge of signs and symptoms of STI with proportion that would be expected for each of them by mere chance. As an alternative to chi-square, when the prerequisites were not met, the Fisher test.

The project was approved by the Research Ethics Committee of the State University of Paraíba (UEPB) under the Certificate of Presentation for Ethical Appreciation (CAAE): 28723614.6.0000.5187. They respected the ethical guidelines contained in Resolution 466/2012, of the National Health Council. Participants signed the Free and Informed Consent Term (FICT) and were assured of secrecy, privacy and the right to decline, at any time investigation, without any kind of burden due to the withdrawal.

## Results

As a result of sociodemographic characteristics, 58 people participated, of which 30 (52%) were men and 35 (60.4%) of the participants were between 41 and 60 years of age. With regard to schooling, 20 (34.5%) completed High School and 19 (32.7%), Higher Education. The majority had a religious belief (93.1%), reported income of one minimum wage (65.5%) and had no partner (65.5%).

Table 1 presents the health and prevention aspects of STIs. It was found that men presented higher frequency of alcohol consumption (p <0.001) and illicit drugs (p = 0.006). Among the findings, men used a male condom more frequently than women using a female condom (p = 0.003), although they had a higher self-reported knowledge of the prevention of STIs (p = 0.006). Television was the most frequent means for obtaining information by women (p <0.001), while internet access was higher in men (p <0.001).

**Table 1:** Characterization referring to health and prevention of STIs * in blind people. Campina Grande, PB, Brazil, 2016

Variables		Sex				p‡
		Men		Women		
		n†	%	n†	%	
Drinking						
	Affirmed	25	83.0	0	0.0	<0.001§
	Denied	5	17.0	28	100.0	
Use of prohibited drugs						
	Affirmed	7	23.0	0	0.0	0.006§
	Denied	23	77.0	28	100.0	
Condom use						
	Always	8	27.0	0	0.0	0.003§
	Sometimes/Don't use	22	73.0	28	100.0	
Knows ways to prevent						
	Affirmed	23	77.0	28	100.0	0.006§
	Denied	7	23.0	0	0.0	
Means of obtaining information						
Radio						
	Affirmed	11	37.0	26	93.0	<0.001§
	Denied	19	63.0	2	7.0	
Television						
	Affirmed	13	43.0	28	100.0	<0.001§
	Denied	17	57.0	0	0.0	
Internet						
	Affirmed	4	3.0	18	60.0	<0.001§
	Denied	26	97.0	10	40.0	
Others						
	Affirmed	15	50.0	22	79.0	0.023║
	Denied	15	50.0	6	21.0	

* STI = Sexually Transmitted Infections;

† n = absolute number;

‡ *p =* statistically significant values p<0.05;

§ *p* = Fisher's test;

║ *p* = chi-square test

Table 2 shows the aspects of sex life and frequency of STIs. Among the participants, there was affirmation of active sexual life and some were affected by a Sexually Transmitted Infection (STI).

**Table 2:** Frequency of sexual life and STI in blind people. Campina Grande, PB, Brazil, 2016

Variables		n†	%
Sex life			
	Active	34	58.6
	Not active	23	39.7
	Not informed	1	1.7
STI*			
	Has had one	21	36.2
	Has never had one	37	63.8
Type of STI* they contracted			
	*Trichomonas vaginalis*	11	52.4
	*Neisseria gonorrhoeae*	5	23.8
	Human Papillomavirus	1	4.8
	Did not know how to report	4	19.0

* STI = Sexually Transmitted Infections;

† n = absolute number

As shown in Table 3, after the intervention, the increase in knowledge about signs and symptoms of STI was detected, and knowledge was verified on all variables above the quartile 75.

**Table 3:** Knowledge of blind people about signs and symptoms of STIs * before and after educational intervention. Campina Grande, PB, Brazil, 2016

Signs and symptoms of STI *	Before†				After‡			
	Knew		Did not know		Knew		Did not know	
	n§	%	n§	%	n§	%	n§	%
Burning or painful urination	47	81.0	11	19.0	52	89.7	6	10.3
Pain during sexual intercourse	41	70.7	17	29.3	57	98.3	1	1.7
Pain in the pelvic or abdominal region	36	62.1	22	37.9	53	91.4	5	8.6
Bullous or ulcerated lesions in the mouth	37	63.8	21	36.2	58	100.0	0	0.0
Purulent secretions in the penis, anus or vagina	43	74.1	15	25.9	58	100.0	0	0.0
Blisters, warts or ulcerations on the genitals	46	79.3	12	20.7	56	96.6	2	3.4
Enlargement of the ganglia of the inguinal region	24	41.4	34	58.6	51	88.0	7	12.0

* STI = Sexually Transmitted Infections;

† Before (x2 = 29.14; p < 0.001);

‡ After (p = 0.005);

§ n = absolute number

When considering the before and after the educational intervention, participants' knowledge was improved, with emphasis on infertility and chronic pelvic pain, which reached 93% and 86%, respectively, according to data from Table 4.

**Table 4:** Knowledge of blind people about clinical complications of STIs*, before and after educational intervention. Campina Grande, PB, Brazil, 2016

Clinical complications of STIs*	Before†				After‡			
	Knew		Did not know		Knew		Did not know	
	n§	%	n§	%	n§	%	n§	%
Infertility	42	72.4	16	27.6	54	93.0	4	7.0
Chronic Pelvic Pain	34	58.6	24	41.4	50	86.0	8	14.0
Cancer	40	69.0	18	31.0	48	83.0	10	17.0
Abortion	39	67.2	19	32.8	44	76.0	14	24.0
Congenital infections	33	56.9	25	43.1	43	74.0	15	26.0
Facilitates HIV infection║	34	58.6	24	41.4	39	67.0	19	33.0

* STI = Sexually Transmitted Infections;

† Before (x^2^ = 5.37; p < 0.371);

‡ After (p = 0.008);

§ n = absolute number;

║ HIV = Human Immunodeficiency Virus

After the educational intervention, it was also verified that the STIs with the highest percentages of knowledge were: AIDS, syphilis and gonorrhea (100%), according to data shown in Table 5.

**Table 5:** Knowledge of blind people on STIs*, before and after the educational intervention. Campina Grande, PB, Brazil, 2016

STI*	Before†				After‡			
	Knew		Did not know		Knew		Did not know	
	n§	%	n§	%	n§	%	n§	%
Hepatitis B	6	10.3	52	89.7	47	81.0	11	19.0
AIDS	57	98.0	1	2.0	58	100.0	0	0.0
Syphilis	38	66.0	20	34.0	58	100.0	0	0.0
Soft cancer	23	39.7	35	60.3	49	84.0	9	16.0
Herpes	22	37.9	36	62.1	49	84.0	9	16.0
Donovanose	6	10.3	52	89.7	36	62.0	22	38.0
Trichomoniasis	29	50.0	29	50.0	55	95.0	3	5.0
Chlamydia	5	8.6	53	91.4	44	76.0	14	24.0
Gonorrhea	45	77.6	13	22.4	58	100.0	0	0.0

* STI = Sexually Transmitted Infections;

† Before (x^2^ = 191.50; p < 0.001);

‡ After (p < 0.001);

§ n = absolute number

## Discussion

The sociodemographic characteristics of the participants in this study differ from those found in another study carried out in the city of Uberaba, Minas Gerais, Brazil, with 33 people with visual impairment, in which the highest prevalence was found among women and most retirees, but they are similar regarding the educational level^(^ ^16^ ^)^.

The association found in this study between sex and variables related to health and STI prevention corroborates another investigation with blind people in which an association between STI-related sexual practice and sex was identified. However, this same research did not identify an association between sex and knowledge about STI prevention and transmission, a fact that can be explained by the small sample size (n = 36)^(^ ^17^ ^)^.

The age range of the majority of the participants in this study classifies them as productive working age, since Brazilian labor legislation considers productive work for men up to 65 years of age and women up to 60 years of age. Even in the economically active age, a significant portion is unemployed, among those who work, the majority are employed. However, a socializing force that impels them to the study valorization as an incentive to the development and the personal autonomy, to the proactive way of existential and social transformation whose support comes from the legislation emanating of the State that, among other aspects, assures the supply of technologies assistance to enhance the functional skills of people with disabilities and to promote independent living with social inclusion.

The participants' religiosity profile indicates, mainly, the relationship with some religious creed. It is a salutary practice in which, in detriment of the difficulty in evaluating the influence of spirituality in the improvement of health, people find in prayer, meditation and/or reflection, ways of obtaining greater physical, psychological and social comfort^(^ ^18^–^19^ ^)^, so that personal / spiritual well-being and self-esteem improve the quality of life^(^ ^20^ ^)^.

Regarding income, the majority of participants received a minimum wage, indicating that they worked and / or were entitled to the Continuous Benefit Benefit, which ensures the monthly benefit for the disabled person who does not have the means to provide for their own subsistence. In order to minimize the difficulty of inclusion in the labor market, the Brazilian State normalized that companies with 100 or more employees should hire people with disabilities and rehabilitated, in the following proportion: up to 200 employees, 2%; from 201 to 500, 3%; from 501 to 1000, 4%; from 1001 onwards, 5%^(^ ^21^ ^)^.

In relation to the conjugal experience of the majority of blind people lived with no partner, which can be explained by comparing these results with those of a study conducted in Feira de Santana, Bahia, Brazil, with eleven blind people, of both sexes, aged between 22 and 54 years, in which, according to participants' complaints, the researchers concluded that blind people remain mostly unmarried, invisible and vulnerable, with the need for sex education as a path to social inclusion^(^ ^7^ ^)^.

In addition, unseen women are perceived as inhabiting a body that dissociates from the current social prototype, which makes them discredited for the role of caregivers, wives and mothers^(^ ^9^ ^)^. In this sense, in a study carried out in Nepal, with pregnant and disabled women, the participants reported embarrassment in exposing their own body, a barrier to the demand for care. In turn, health professionals felt unprepared to meet the health needs of pregnant women with disabilities^(^ ^22^ ^)^.

Regarding the sexual and reproductive health of blind people, it was observed in this study that 36.2% of the participants reported involvement due to Sexually Transmitted Infections. It should be added that the lack of awareness of some signs and symptoms of STI and its clinical complications may be related to possible failures in the health care of blind people, which would justify the carrying out of health education activities with the purpose of instrumentalizing people with the necessary guidelines for safe sexual practice, also guaranteeing the application of public policy.

In Brazil, although there are policies focused on the sexual and reproductive rights of people with disabilities, attitudinal barriers undermine social participation. This context has deserved researchers attention to the need for validation of assistive technologies adapted to people with visual impairment^(^ ^11^ ^)^ and aimed at health education in primary care, school or other environment, with a view to sensitizing visually impaired women for the use of the female condom^(^ ^23^ ^)^.

According to the data of this investigation, the information obtained on blind STI / Aids came from a variety of sources, such as radio and television, highlighting women who reached the highest percentages in all media. It also highlighted the existing gap in relation to obtaining knowledge offered by health professionals. These same communicative tools were found in study with visually impaired people in Ethiopia, Kenya, South Africa, Mozambique, Rwanda and Uganda. The results demonstrated that many health professionals do not have the necessary skills to respond adequately to the needs of blind people^(^ ^24^ ^)^, which do not feel covered by public health policies or included in the various programs, such as the prevention of STI/HIV/AIDS^(^ ^7^ ^)^.

In addition, when it comes to the vulnerability of the participants in this study, the consumption of alcohol or illicit drugs can lead to risky sexual behaviors, such as low condom use. This relationship was strengthened by a study report in which, in the female sex, associations were identified for seropositivity and consumption of drugs, alcoholic beverages and other drugs; being married or in stable union. In the male sex, there were associations of seropositivity with consumption of other drugs and homosexual/ bisexual orientation^(^ ^25^ ^)^.

These situations predispose to exposure to STIs and / or unplanned pregnancy. In addition, in this study, non-use of condoms can be explained by data from another survey of 3,482 people of both sexes, whose researchers also detected behaviors vulnerable to STI contamination due to condom disuse, for several reasons: 280 (11%) do not like to use a condom; 716 (28.1%) because they knew the partner; 36 (1.4%) because the partner did not accept; 1,044 (41%) because they used another contraceptive method and 294 (11.5%) for other reasons^(^ ^26^ ^)^.

Although the women in this study claimed to have unprotected sex, they all knew the ways of preventing STIs, but considerable numbers of men were unaware of them. It is understood that misinformation in the context of sexual health entails limitations for people, since they require specific components for access to information^(^ ^11^ ^)^, in addition to expanding the possibilities of contracting an STI. STIs, such as *Trichomonas vaginalis, Neisseria gonorrhoeae* and Human Papillomavirus, reported by the participants, are considered a global public health problem^(^ ^27^ ^)^.

In Brazil, a retrospective clinical and laboratorial evaluation of 4,128 patients in a specialized STI center revealed that these are predominant in 76% of men. The occurrence of these infections was higher in the 20-29 age group^(^ ^1^ ^)^, differing from a study in South Korea where the incidence was higher in women aged 60 years or older. However, the incidence for men increased from 23.7 in 2009 to 15.7 per 100,000 in 2014, with a marked reduction in groups of 40 years or more of this population^(^ ^27^ ^)^. A study carried out in Shanghai reports that, in addition to the incidence of *Neisseria gonorrhoeae* being the highest of bacterial STIs, it can produce severe complications as well as emerging resistance to third generation cephalosporins, allowing the emergence of a potentially intractable gonorrhea era^(^ ^28^ ^)^.

Participants' knowledge about STIs and clinical signs and symptoms before the educational intervention reached unsatisfactory percentages. Likewise, in relation to the possible complications resulting from STIs, it is considered that the preliminary percentages to the intervention also indicated considerable ignorance. However, at this stage of the study, the participants' education incorporated some of the knowledge socialized by the scientific literature about STIs presenting risks to sexual and reproductive health. This finding may be related to the study site, since the ICCG is configured as a reference educational institution for blind people, offering qualification for students enrolled in activities.

Thus, after the health intervention, participants' answers demonstrated an understanding of STIs in a more eloquent and well-founded way, but at the same time, they showed the need for constant education and attention in sexual and reproductive health, since only the STIs, Aids, syphilis and gonorrhea obtained the total percentage of positive responses.

The results of the educational intervention resemble those of a study conducted in Bangladesh, which describes the main indicators of women's reproductive health, through learning and participatory action. Researchers have improved women's knowledge about sexual health, morbidity, and ways to prevent and treat STIs. However, they argue that the limited shift in health practices may stem from the rural sociocultural context of Bangladesh in which women may face limited health services to act on foreground^(^ ^29^ ^)^.

From this perspective, it was evidenced that the educational practice, mediated by the problematizing pedagogy, presented potential of modification of the knowledge of blind people in a way that these sought reflections from stimuli to solve problems^(^ ^13^–^15^ ^)^. This characteristic is limited to the educational intervention used as innovative health technology, which used a consolidated educational trend for a blind public to reflect on a theme considered taboo in this social segment. Therefore, the study is relevant to the scientific knowledge divulged, representing progress in the subject that, in recent years, was object of another study, in which it was tried to make accessible the knowledge about STI for blind people through the validation of educational text^(^ ^11^ ^)^.

The limitations of this study are related to the use of variables with self-reported responses, which allows memory bias, the impossibility of performing a larger quantitative of educational meetings, due to the mobility difficulties experienced by the participants, as well as having analyzed only the acquisition of knowledge, to the detriment of behavior change.

It is considered that this study has the potential to contribute to the nursing actions in the field of sexual health education for people with disabilities, since the characterization of the vulnerability situation of these people enables health managers and professionals to plan strategies and actions of prevention in line with the social and health reality of these individuals.

## Conclusion

The results indicate that, before the educational action, the participants' knowledge about STIs was marked by misunderstandings, inadequacies and lack of knowledge, especially in the prevention of these infections. After the educational intervention, in the light of problematizing pedagogy, knowledge about STIs, signs, symptoms and possible complications was (re)constructed, empowering blind people to adopt preventive practices. Thus, the educational intervention was effective to intensify knowledge about STIs, and the more informed people were, the better adherence to preventive health measures.

The fact that the participants did not confirm the information obtained from health professionals about STIs indicates that this issue related to people with disabilities needs permanent education and a transversal approach in the curricular components, aiming at the demystification of prejudices that reinforce the vulnerability of these people, compromising the experience of sexuality and the preservation of sexual and reproductive health. It is necessary that nurses devote greater attention to this population group, which has insufficient basis for the care with sexual health, as it faces social barriers that make difficult the search for knowledge regarding health promotion.

In this perspective, it is suggested that other studies approach the same subject, aiming to evaluate the attitudes and practices of people with disabilities after health education, in order to verify the behavioral modification regarding the participant's sexual and reproductive health.
